# Case of Destruction of the Lower Jaw and of a Portion of the Face

**Published:** 1852-01

**Authors:** Frank H. Hamilton

**Affiliations:** one of the Surgeons to the Buffalo Hospital.


### ARTICLE XI.

*Case of Destruction of the Lower Jaw and of a portion of the Face, under Homeopathic Treatment; also the Result of a Novel Operation made for the Restoration of the Lower Lip, with some remarks on the Formation of New Skin.* BY FRANK H. HAMILTON, one of the Surgeons to the Buffalo Hospital.

MARTIN NEUMAN, seven years old, was attacked on the 10th of August, 1849, with a mild dysentery. The family were German and sent for a German homeopathist, who gave him at once small pills, which "looked and tasted like sugar!" also a powder and solution.

Within seven days from the time the medicines were commenced salivation began, and small ulcers appeared upon the inside of the mouth, upon the gums, &c. Three days after, the ulceration had extended so rapidly that the lower lip was nearly separated, and in a day or two more it fell off entirely. Three months later, the greater portion of the lower jaw came away in one piece, being two and a half inches long and including the whole diameter of the bone with its corresponding teeth. The bone and teeth are now in my possession.

It is a coincidence somewhat remarkable, that the sister Amelia, several years older, was ill in the same way, and at the same time, (it was during the prevalence of the cholera in this city,) and took medicines from the same man, viz. solutions, &c., and within one week she was severely salivated also, and her mouth became ulcerated, but no destruction of bone or of the soft parts ensued.

In January, 1850, the lad was brought to me, by his father. The lower jaw was then reproduced through the whole extent of that which had been destroyed, but the teeth were of course not replaced: nor was there a vestige of a lower lip, and even the bone was thinly and imperfectly covered with integument. His condition was distressing in the extreme, since he could masticate only with great difficulty, and his saliva was constantly pouring upon his chin, excoriating his face and neck, and saturating his clothes.

*First operation for the restoration of the lip.* January 14th, 1850, in the presence of the class at the medical college I abraded the upper edge of the skin corresponding to the lower lip, to the extent of a quarter of an inch each way from the center; from either extremity of this horizontal incision I cut perpendicularly about one inch, and then starting from the lower end of these incisions, I carried the knife outward and downward to the left, and outward and downward to the right, one inch and a half. The two lateral pieces thus marked out, were now dissected from the jaw and slid upward and drawn together with sutures, above the central piece; the lower edge of the lateral pieces thus united were stitched also to the upper and abraded edge of the central piece.

The object in leaving a central piece attached to the jaw, and uniting the lateral pieces above it, was to prevent the lateral pieces, which were to constitute the new lip, from drawing down again by the contraction of the wound below. The plan was original, I believe, and proved successful. The new lip, however, became, in process of time, through stretching and shrinking, insufficient, and I made a second operation to increase the depth of the lower lip, and prevent more effectually the saliva from dribbling from the mouth.

*Second Operation*, August 28th, 1850, at my office, in presence of Drs. Samuel Carey, Camp, and others. My mode of procedure was entirely new, and as I believe, has established an important principle in this class of operations. The operation was as follows: a single incision was made just under the chin, extending along the lower edge of the inferior maxilla, about three inches from side to side. All the integument comprised between this horizontal incision and the upper edge of the lower lip, was now raised from the bone, and the entire mass was slid upward until its lower edge was made to correspond with a line just below the upper border of the jaw. Here this edge was made fast to the *periosteum*, by several interrupted sutures. The gaping wound below was left to close by granulation. The result has been, that adhesion occurred between the lower edge of the flap thus secured, and the periosteum, and no disposition was afterwards shown in the flap to draw downward as the wound cicatrized; but on the contrary, the skin from below, that is, from under the chin and the neck, was somewhat drawn upward, and thus between the formation of new skin and contraction of the skin from below, the wound closed.

The new principle established, is, that *by attaching the skin directly to the* PERIOSTEUM *its displacement by cicatrization, and contraction is prevented.* Every one who has operated for the restoration of the lower lip, will see the advantages which this plan offers. There is nothing to which the upper, free border of the new lip can be attached, and there is, consequently, nothing but the mere transverse tension of the lip, to prevent its descending as cicatrization progresses below. This tendency I sought to avoid in the first operation by leaving a central piece untouched and adherent to the bone, and then bringing the new lip above it. But this procedure requires a sacrifice of a portion of the transverse diameter of the lip, and is often wholly inadmissible, and always objectionable, if the same end can be attained by another mode. This new mode, as we have demonstrated, prevents the sliding downward, without sacrificing any portion of the lip. These remarks are applicable especially to cases of *complete* loss of the lip. Where only a portion is lost, various other methods of supplying the deficiency may be practiced; as by stretching the lip, or sliding from the cheeks, or even by an operation of "torsion" from the cheeks.

This idea originated in having observed elsewhere the capacity of periosteum to form skin. I have several times proved, contrary to the often repeated doctrine, that skin may form *de novo*, independent of old skin: as where there has been an extensive destruction of the integuments over a bone—where the parts have been torn away or have sloughed quite to the periosteum, and, consequently, no old skin could have been left from which the new could form, except at the edges; yet in the very center of this broad ulcer, new skin has sprung up like an oasis, and gradually spread outward in all directions. But this has always been where the periosteum was actually exposed, which first becoming white and spongy, has soon shown itself to be the nucleus of a new skin—in fact, it has become ***itself converted into skin***, remaining ever afterwards depressed, immovable and adherent to the bone at that point.

The result of the case of the lad Neuman, is, that he has a lower lip, sufficient to cover the gums and a part of the bodies of a set of artificial teeth which our ingenious dentist, Dr. Harvey, has made for him. The lip is narrow, for we have not yet been able to prevent the contraction and rolling in of the upper edge, as it heals, but it would certainly have been much narrower, or entirely lost if the adhesion to the periosteum had not bcen effected.

I will not omit to say, that by the constant effort to use the lower lip, or perhaps simply by the lapse of time, the lip has very perceptibly lengthened in its vertical diameter during the last six months.

